# SPOCK1 Overexpression Suggests Poor Prognosis of Ovarian Cancer

**DOI:** 10.3390/cancers15072037

**Published:** 2023-03-29

**Authors:** Lóránd Váncza, Anna Horváth, Lee Seungyeon, András Rókusz, Katalin Dezső, Andrea Reszegi, Gábor Petővári, Martin Götte, Ilona Kovalszky, Kornélia Baghy

**Affiliations:** 1 Department of Pathology and Experimental Cancer Research, Semmelweis University, 1085 Budapest, Hungary; 2 Department of Pathology, Forensic and Insurance Medicine, Semmelweis University, 1085 Budapest, Hungary; 3 Department of Gynecology and Obstetrics, University Hospital Münster, 48149 Münster, Germany

**Keywords:** SPOCK1, ovarian cancer, testican-1, proteoglycan, extracellular matrix

## Abstract

**Simple Summary:**

The SPOCK1 proteoglyan has been discovered as an oncogene with overexpression, promoting tumor formation and progression, and linked with poor survival rates. However, information on SPOCK1′s significance in ovarian cancer is limited. The purpose of this study was to explore the role of SPOCK1 in ovarian cancer. With that goal, two ovarian cancer cell lines, as well as tissue and serum samples from patients suffering from ovarian cancer, were studied. The proteoglycan was overproduced in cell lines upon the addition of artificial SPOCK1 vector. As a result, SPOCK1 overexpressing cells increased their rate of cell division and migration. In line with this, ovarian cancer tissues and blood samples exhibited higher SPOCK1 levels than healthy controls. Furthermore, SPOCK1 levels in untreated ovarian cancer serum and tissue samples were higher than in chemotherapy-treated patients. Our results indicate that SPOCK1 may serve as a therapeutic target and could be utilized for monitoring ovarian cancer.

**Abstract:**

Purpose: Sparc/osteonectin, cwcv, and kazal-like domains proteoglycan 1 (SPOCK1) has been found in a variety of malignant tumors and is associated with a poor prognosis. We aimed to explore the role of SPOCK1 in ovarian cancer. Methods: Ovarian cancer cell lines SKOV3 and SW626 were transfected with SPOCK1 overexpressing or empty vector using electroporation. Cells were studied by immunostaining and an automated Western blotting system. BrdU uptake and wound healing assays assessed cell proliferation and migration. SPOCK1 expression in human ovarian cancer tissues and in blood samples were studied by immunostaining and ELISA. Survival of patients with tumors exhibiting low and high SPOCK1 expression was analyzed using online tools. Results: Both transfected cell lines synthesized different SPOCK1 variants; SKOV3 cells also secreted the proteoglycan. SPOCK1 overexpression stimulated DNA synthesis and cell migration involving p21^CIP1^. Ovarian cancer patients had increased SPOCK1 serum levels compared to healthy controls. Tumor cells of tissues also displayed abundant SPOCK1. Moreover, SPOCK1 levels were higher in untreated ovarian cancer serum and tissue samples and lower in recipients of chemotherapy. According to in silico analyses, high SPOCK1 expression was correlated with shorter survival. Conclusion: Our findings suggest SPOCK1 may be a viable anti-tumor therapeutic target and could be used for monitoring ovarian cancer.

## 1. Introduction

Ovarian cancer is a common form of malignant tumor in female patients, typically appearing after menopause. Germline or acquired mutations of the BRCA1 and BRCA2 genes results in the impairment of their proteins that are involved in the homologue recombination of DNA [1]. Among other heritable cancers, Lynch syndrome can also facilitate the development of ovarian cancer through missense mutations in mismatch repair genes [2]. However, the majority of ovarian tumors arise without inherited genetic mutations. Often, the early symptoms of ovarian cancers are mild, resulting in delayed diagnosis and shorter life expectancy [3]. In the case of heritable familial malignancies, regulated monitoring aids in early tumor detection, whereas tumor markers indicating the potential risk may support the early discovery of cancers that are not inherited. One of the best-known markers is CA125, which is positive in the presence of epithelial ovarian cancers, such as serous, mucinous, or clear cell carcinomas. This mucin-like protein, found on the surface of tumor cells, may play a role in galectin-1 export [4]. Although CA125 has a high level of specificity, its sensitivity, alone or in conjunction with other circulating markers or diagnostic tools, may not be sufficient to detect early ovarian cancers [5]. Early symptoms, such as abdominal bloating, sensitivity of the pelvic region, constipation, etc., might be subtle or deceptive [6]. Thus, searching for new potential markers whose expression increases in parallel with the tumor development could improve outcomes of this life-threatening malignancy.

SPOCK1/Testican-1 is a heparan sulfate/chondroitin sulfate proteoglycan, considered as a resident of the extracellular matrix [7]. In 1992, when the proteoglycan was isolated from the seminal plasma, it was given the name testican. Five years later, it was cloned from the central nervous system as a component of neural synapses [8]. Over time, it was also found in the neuromuscular junctions, the endothelial cells, and the extracellular matrix. Contradictory data have been published regarding its ability to stimulate or inhibit various metalloproteases [9]. Its oncogenic potential was first documented in 2011 [10,11]. Since then, its potential to promote tumor growth has been reported in nearly all types of epithelial cancers [12]. Despite these findings, testican is still regarded as an extracellular matrix protein, secreted to the tumor stroma by the stromal cells, such as endothelial cells and tumor-associated fibroblasts [13]. It inhibits tumor cell apoptosis and, with the cooperation of TGF-β, facilitates EMT [14]. Recent studies have demonstrated its localization in the nuclei and mitochondria of tumor cells, but its physiological functions have not yet been determined [15]. Its physiological functions in epithelial cells have yet to be discovered, with the exception of a single publication about its implication in heat regulation of fat cells. SPOCK1 has been detected in the blood circulation of patients with sepsis [16]. Our recent studies on mouse, rat and human livers revealed that experimental hepatocarcinogenesis upregulates testican-1 in the transformed hepatocytes, and that abundant amounts of the proteoglycan can be detected in liver cirrhosis and hepatocellular cancer, but not in the ECM [15]. Additionally, in vitro experiments revealed that the proteoglycan has a signal for secretion, allowing it to leave the cells and enter the culture medium, thereby maintaining the equilibrium inside the cells. Given the current state of knowledge, it is plausible that testican-1 accumulated in tumor cells could be secreted and enter the blood circulation. If this hypothesis can be confirmed, testican-1 could be utilized as a new tumor marker.

High levels of testican-1 have also been detected in ovarian cancer tissues and cell lines. Its knock down by shRNA inhibited proliferation and invasion of tumor cells [17] and downregulated MAPK and Akt signaling. These results support our presumption that SPOCK1 is a protein that could be used as an auxiliary tumor marker for ovarian cancers.

To demonstrate this possibility, ovarian cancer-derived cell cultures and human ovarian tumor specimens were tested, in combination with monitoring the proteoglycan in the blood circulation.

## 2. Materials and Methods

### 2.1. Materials

All reagents and materials were purchased from Merck KGaA (Darmstadt, Germany) unless indicated otherwise. Antibodies used in the present study are listed in Supplementary Table S1.

### 2.2. Cell Cultures

Ovarian cancer cell lines SKOV3 and SW626 were donated by Professor Dr. Martin Götte (University of Münster, Germany). SKOV3 cells were cultured in McCoy’s 5a (Modified) Medium (Catalog No. 30-2007) supplemented with 10% fetal bovine serum (FBS; FB-1001B/500, Biosera, Kansas City, MO, USA), 100 unit/mL of penicillin, and 100 μg/mL of streptomycin (P0781-100ML). For SW626 cells, Leibovitz’s L-15 Medium (Catalog No. L15-XA) was supplemented with 10% fetal bovine serum (FBS; FB-1001B/500, Biosera, Kansas City, MO, USA), 2 mM of L-glutamine (XC-T1715/100; Biosera), 100 unit/mL of penicillin, and 100 μg/mL of streptomycin (P0781-100ML) and Na-bicarbonate (S8761).

### 2.3. Transfection

The construction of plasmid for wild-type and SPOCK1 overexpressing vectors has been described previously [15]. The transfection was carried out with the Neon Transfection System (MPK5000, Life Technologies, Carlsbad, CA, USA), using a 100 µL pipette tip. In the process, 2 × 250,000 SKOV3 cells and 2 × 50,000 SW6262 cells were used with 10 µg of SPOCK1 pcDNA™4/TO and empty vector pcDNA™4/TO. Electroporation was then performed with the following settings according to manufacturer’s instructions: in the case of SKOV3, 1170 V, 30 ms, and 2 pulse, while for the SW626 cell line, 1100 V, 30 ms, 1 pulse was used. Cells were seeded in 6-well plates in culture medium supplemented with 20% FBS and 2 mM of L-glutamine. In order to identify the sub-population of transfected cells and to determine the transfection efficiency, a complete growth medium supplemented with 500 µg/mL of Zeocin was applied.

### 2.4. Immunocytochemistry

Cells were plated on sterilized coverslips in 6-well plates (2 × 200,000 cell/well) and a complete growth medium was added. After 48 h, the cells were rinsed briefly three times with phosphate-buffered saline (PBS; containing 137 mM of NaCl, 2.7 mM of KCl, 10 mM of Na_2_HPO_4_, and 1.8 mM of KH_2_PO_4_, pH 7.5) and were incubated with methanol (−20 °C) for 10 min. Fixed cells were rinsed three times with PBS and blocked with 5% *w*/*v* bovine serum albumin (BSA) in PBS. Incubation with SPOCK1 and p21^CIP1^ primary antibodies dissolved in 5% *w*/*v* BSA (PBS) was performed at 4 °C overnight. Next, coverslips were washed three times with PBS and exposed to fluorescent-labeled Alexa Fluor 568-conjugated secondary antibodies for 40 min at room temperature, followed by staining with DAPI (1:50; D9542). After washing, the cells were mounted using Fluoromount (F4680; Merck KGaA, Darmstadt, Germany).

### 2.5. Automated Western Blotting (WES)

Once reaching 70–80% confluency, cells were harvested in lysis buffer containing 20 mM Tris, 150 mM NaCl, 2 mM EDTA, 0.5% Triton X-100 supplemented with protease (P8340, Merck KGaA, Darmstadt, Germany) and phosphatase inhibitors. Using Protein Assay Dye Reagent Concentrate (500-0006, Bio-Rad, Hercules, CA, USA), the concentration was determined. In control and SPOCK1-transfected cells, SPOCK1, GAPDH and p21^CIP1^ protein levels were immunodetected with WES™ Simple analysis on the WES™ system (ProteinSimple-Biotechne 004–600). The 12–230 kDa Separation Module (ProteinSimple SM-W004) and, depending on the primary antibodies, the Anti-Mouse (ProteinSimple DM-002) or Anti-Rabbit Detection Module (ProteinSimple DM-001) were applied.

### 2.6. BrdU Incorporation Assay

Culture medium was replaced with fresh medium supplemented with 10 µM BrdU (B5002, Merck KGaA, Darmstadt, Germany) and was incubated for 30 min at 37 °C. Ice-cold methanol was used for 10 min to fix the cells. After washing with PBS for 3 × 5 min, cells were incubated with 2N HCl for 10 min at room temperature. Cells were again washed three times, then incubated with 0.2% Triton X-100 to improve the penetration of the Anti-BrdU antibody. After one hour of incubation at room temperature the cells were washed and labeled with Alexa Fluor 488 and DAPI (1:200). The cells were mounted using Fluoromount.

### 2.7. Migration Assay

Control and SPOCK1-transfected SKOV3 and SW626 cells were plated onto a round-bottomed 6-well culture plate (standard plate) in culture medium supplemented with 10% FBS. From each cell line, 2 × 10^5^ cells/well were transferred and then left until they reached 90–95% of confluence. To eliminate the contribution of cell proliferation to gap closure, 2 h of incubation with mitomycin C was applied. A scratch was made with a 200 µL pipette tip to create an incision-like gap. Cells were fixed at 0, 48, 72 and 96 h time points using methanol and then stained with H&E. The slides were scanned with a Pannoramic P1000 scanner (3DHISTECH Ltd., Budapest, Hungary) or photographed using an optical camera.

### 2.8. Human Materials

Plasma samples of the peripheral blood of 67 adult patients were selected to analyze SPOCK1 levels. The study was approved by and registered at the Institutional Review Board, Semmelweis University Regional and Institutional Committee of Sciences and Research Ethics (ETT-TUKEB: IV/2734-3/2022/EKU). Informed consent was obtained from each patient. Patients’ age varied between 44 and 84 years, and the diagnosis for all patients was malignant tumor of the ovary (SNOMED: 363443007). Raw data of SPOCK1 plasma levels are provided in Supplementary Table S3. The extraction of plasma from peripheral blood samples was carried out within 24 h of sampling by centrifugation at 1800× *g* for 10 min and then plasma was stored at −80 °C.

Twenty-five human tissue samples were collected from the biopsy archive of the Department of Pathology and Experimental Cancer Research, Semmelweis University. Ethical approval to collect surgical materials was obtained from the Institutional Review Board, Semmelweis University Regional and Institutional Committee of Sciences and Research Ethics (TUKEB permit number: 155/2012), Medical Research Council Committee of Science and Research Ethics (permit number: 61303-2/2018/EKU).

### 2.9. Immunhistochemistry

Formalin fixed paraffin-embedded (FFPE) human ovarian tissues were sectioned and immunostaining was performed. After dewaxing and subsequent rehydration, heat-induced antigen retrieval was achieved by using Dako Target Retrieval Solution, Citrate pH = 6 (S236984–2 (Dako Omnis), Agilent Technologies, Santa Clara, CA, USA). To quench endogenous peroxidases, sections were incubated with 10% H_2_O_2_ solution dissolved in methanol for 20 min. Slides were then washed with PBS for 3 × 5 min. Novocastra Protein Block (RE71102, Leica Biosystems, Wetzlar, Germany) was applied for 10 min to block nonspecific binding.

After washing, slides were incubated with the SPOCK1, Ki67 or CHD1L primary antibody overnight at 4 °C. The next day, sections were thoroughly washed and incubated with the HISTO-Labeling System for 30 min at room temperature (30011.R500, Department of Immunology and Biotechnology, Pécs, Hungary). Diaminobenzidine 1:50 (ImmPACT DAB, SK-4105, Vector Laboratories, Burlingame, CA, USA) was used to visualize the reactions, and hematoxylin counterstaining was performed afterwards. Stained sections were dehydrated and mounted under glass coverslips with BioMount (BMT-500, BIOGNOST D.O.O, Zagreb, Croatia). The slides were scanned with Pannoramic P1000 scanner (3DHISTECH Ltd., Budapest, Hungary). For SPOCK1 immunostaining, evaluation of the staining intensity was performed according to the following scoring scheme: 0: negative, 1: weak, 2: weak–moderate, 3: moderate, 4: moderate-strong, 5: strong.

### 2.10. SPOCK1 ELISA

ELISA was used for quantitative detection of SPOCK1 in the plasma of patients with ovarian cancer (Human SPOCK1/SPOCK/Testican ELISA Kit, Sandwich ELISA, Catalog No. LS-F25604, LifeSpan BioSciences, Seattle, WA, USA) in accordance with the manufacturer’s instructions with a volume of 100 µL per sample. The final optical density values were read with a spectrophotometer. A four-parameter logistic (4PL) curve fitting model was applied in GraphPad Prism^®^ 6 (GraphPad Software, Inc. San Diego CA) to determine the SPOCK1 concentration.

### 2.11. In Silico Analysis

To obtain patient survival data, The Human Protein Atlas database (proteinatlas.org, https://www.proteinatlas.org/ENSG00000152377-SPOCK1/pathology/ovarian+cancer accessed on 20 January 2023) was used, as well as the online Kaplan–Meier plotter tool [18].) For the Human Protein Atlas, the basic settings were not changed, and Kaplan–the Meier curves were saved and presented. In Kaplan–Meier plotter, progression-free as well as overall survival analyses were run. Histology type was set to serous, and the analysis was executed by setting all grades (n = 1104, FDR = 10%); with grade I and II tumors (n = 260, FDR = 1%); and with grade III tumors (n = 798, FDR = 50%).

### 2.12. Statistical Analysis

Statistical analysis was performed and bar charts were drawn using GraphPad Prism v8.01 (GraphPad Software, La Jolla, CA, USA) software. Results were analyzed by unpaired Student’s *t*-test, and significance was defined as * *p* < 0.05, ** *p* < 0.01, and *** *p* < 0.001.

## 3. Results

### 3.1. The Effect of SPOCK1 Level on Survival—In Silico Approach

**Figure 1:**
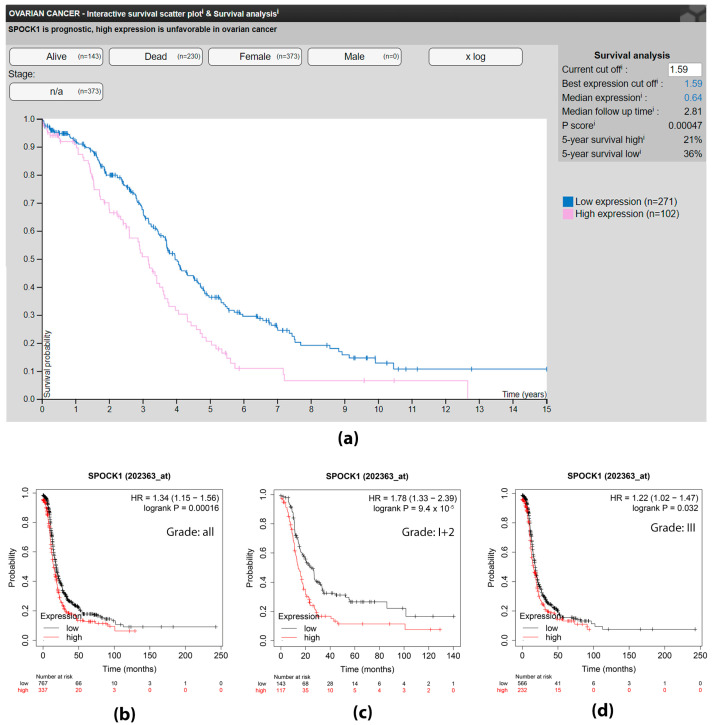
In silico survival analysis of patients with ovarian cancer in the context of SPOCK1 expression levels. (**a**) Survival analysis obtained from the Human Protein Atlas database showing that high SPOCK1 expression is associated with shorter survival (image credit: Human Protein Atlas). (**b**) Progression-free survival analysis by Kaplan–Meier Plotter run on serous tumors of all grades (n = 1104, FDR = 10%); (**c**) with grade I and II tumors (n = 260, FDR = 1%); (**d**) with grade III tumors (n = 798, FDR = 50%).

To determine whether SPOCK1 expression level influences ovarian cancer patient survival, the association between its mRNA level and survival was analyzed using the Human Protein Atlas and Kaplan–Meier plotter (Figure 1).

According to the Human Protein Atlas, SPOCK1 is a prognostic marker for ovarian cancer because its high expression is associated with significantly shorter survival (Figure 1a). Patients with serous ovarian cancer had a significantly shorter survival (Figure 1b), with a modest increase in the hazard ratio (n = 1104, HR = 1.34, FDR = 10%) when analyzed using Kaplan–Meier plotter. However, when tumor grade was considered, high SPOCK1 expression in patients with grade 1 and 2 tumors had a much more pronounced effect on survival, increasing the hazard ratio to 1.78 (Figure 1c, n = 260, FDR = 1%). Surprisingly, in the case of grade 3 tumors, the survival disparity between tumors with low and high SPOCK1 expression nearly disappeared (Figure 1d, n = 798, HR = 1.22, FDR = 50%).

On the basis of these results, it appears that high SPOCK1 mRNA expression has a negative effect on survival, although the extent of this effect appears to vary depending on other factors.

### 3.2. SPOCK1 Localization and Expression in Human Ovary Samples

**Figure 2:**
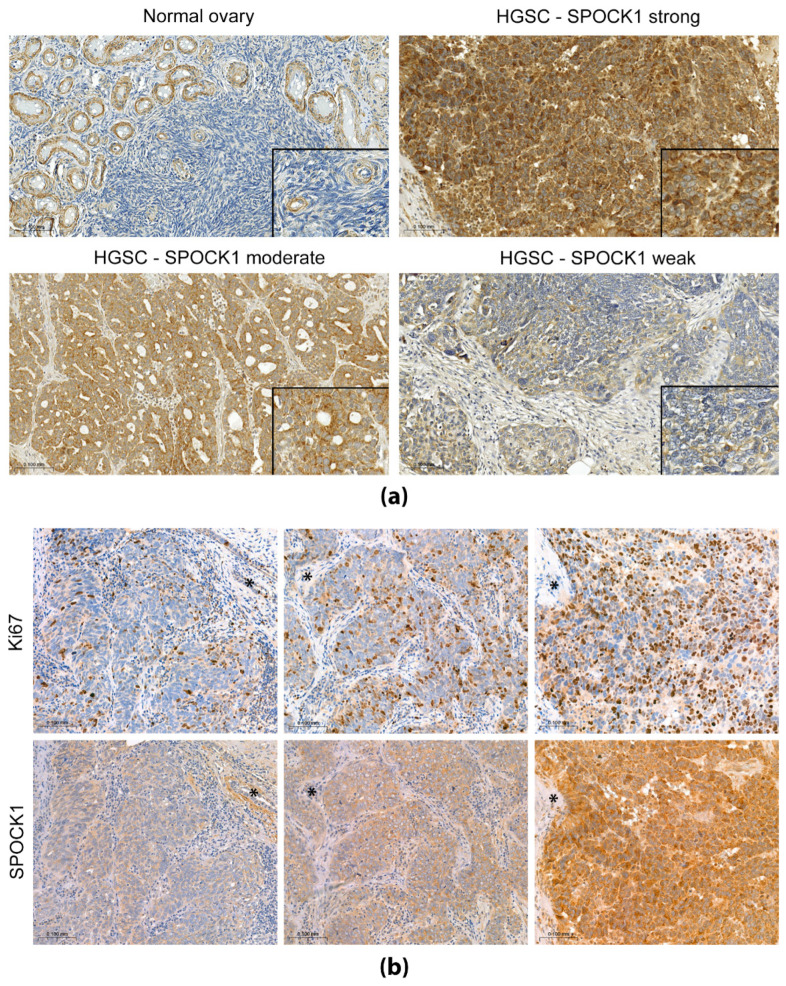
SPOCK1 and Ki67 immunostainings on human tissue samples of normal ovary and high grade serous ovarian carcinomas (HGSC). (**a**) SPOCK1 immunohistochemistry showing strong, moderate or weak staining. Scale bar: 100 µm, and 50 µm for inserts. (**b**) Ki67 and SPOCK1 immunostainings on serial sections to detect their correlation. Asterisks mark the same structures in paired sections. Scale bar: 100 µm.

We examined the expression of SPOCK1 on 25 whole tissue blocks taken from tubo-ovarian high-grade serous carcinomas (HGSC) as well as histologically normal ovaries (Figure 2). Immunohistochemistry revealed that, in normal ovary tissue, SPOCK1 exhibits strong and consistent granular cytoplasmic expression in the smooth muscle wall of vessels and in the hilar smooth muscle bundles. The epithelial cells of epithelial inclusion cysts showed weaker but fairly diffuse SPOCK1 positivity. Ovarian stromal cells were SPOCK1 negative. In tumors, the tumor cells showed diffuse granular cytoplasmic SPOCK1 staining. In most of the tumors, the staining was moderate or strong and formed a homogeneous pattern. In one of the tumors, we saw heterogeneous SPOCK1 expression, where some tumor cell groups showed strong expression, while in other areas, milder expression could be seen. A few samples exhibited weak staining intensity. The consistent positivity of smooth muscle cells in vessel walls served as a positive control. Staining intensities were evaluated by using a 5-part scoring system, and the results are presented in the Supplementary Table S2 for each tissue sample.

Next, Ki67 immunostaining was performed on all tissue samples to discover whether SPOCK1 expression is associated with the proliferation rate. A higher number of Ki67 positive tumor cells were observed in tumors producing abundant SPOCK1. On the other hand, weak SPOCK1 immunostaining was associated with a low number of Ki67 positive cells (Figure 2b).

### 3.3. SPOCK1 Expression in Control and Transfected Tumor Cell Lines

**Figure 3:**
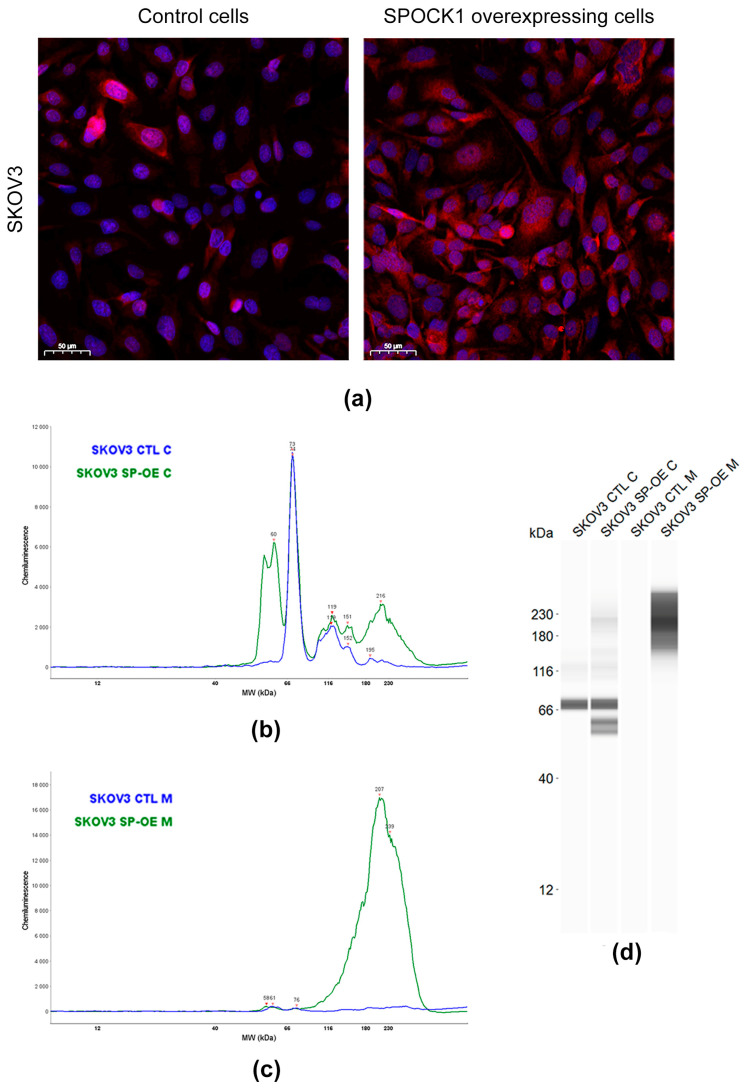
Detecting SPOCK1 level and localization in control and SPOCK1-transfected SKOV3 cells. (**a**) SPOCK1-specific immunostaining of control and SPOCK1 overexpressing cells. WES analysis graphs of SPOCK1 levels in cell lysates (**b**) and supernatants (**c**) from control (CTL) and SPOCK1-transfected (SP-OE) cells. Virtual blot images (**d**) of WES analysis.

**Figure 4:**
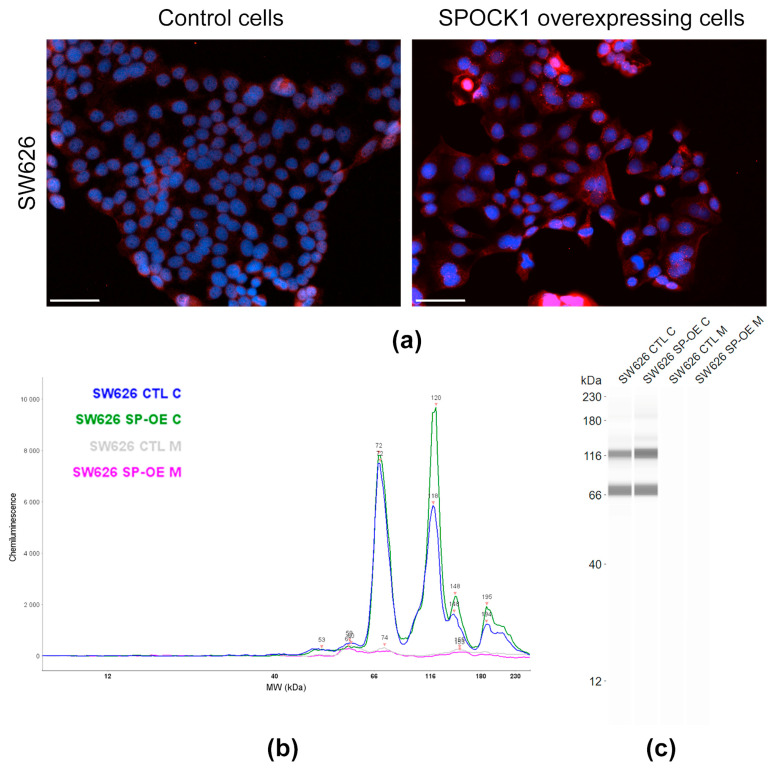
Detecting SPOCK1 level and localization in control and SPOCK1-transfected SW626 cells. (**a**) SPOCK1-specific immunostaining of control and SPOCK1 overexpressing cells. Scale bar = 50 µm (**b**) WES analysis graphs of SPOCK1 levels in cell lysates and supernatants from control (CTL) and SPOCK1-transfected (SP-OE) cells. Virtual blot images (**c**) of WES analysis.

The transfection of both SKOV3 and SW626 cells was successful (Figure 3 and Figure 4). Control SKOV3 cells expressed the proteoglycan in moderate amounts, with a molecular weight of ~73 kDa (Figure 3a–d). Additional peaks were also detected at ~119 and ~152 kDa. No SPOCK1 was detected in their culture media (Figure 3b,c). In SPOCK1-tranfected cells, the excess proteoglycan was observed within the cells, but the majority was secreted into their culture medium. Interestingly, the transfection did not change the size of the ~74 kDa peak, as the same size was seen in control cells. Instead, a new large double peak appeared around ~60 kDa, as well as a ~216 kDa peak, suggesting that the artificially expressed SPOCK1 differs in structure from that which is expressed physiologically. The secreted SPOCK1 had a size peak at ~239 kDa appearing as a “smear” reflecting on a highly glycosylated form of the proteoglycan (Figure 3c,d).

In the case of the SW626 cell line (Figure 4a–c), control cells expressed SPOCK1 with a ~72 kDa and ~118 kDa molecular weight. Smaller fractions were detected at ~148 kDa and ~194 kDa. Their medium was negative for the proteoglycan (Figure 4b,c). Surprisingly, transfection with the same SPOCK1 overexpressing plasmid resulted in no extra peak, but mainly increased the level of the ~118 kDa form. Elevation in the levels of ~148 and ~194 kDa forms upon transfection were also observed (Figure 4b). In contrast to the SKOV3 cells, transfected SW626 cells did not secrete the proteoglycan (Figure 4c,d).

### 3.4. SPOCK1 Overexpression Promotes DNA Synthesis

**Figure 5:**
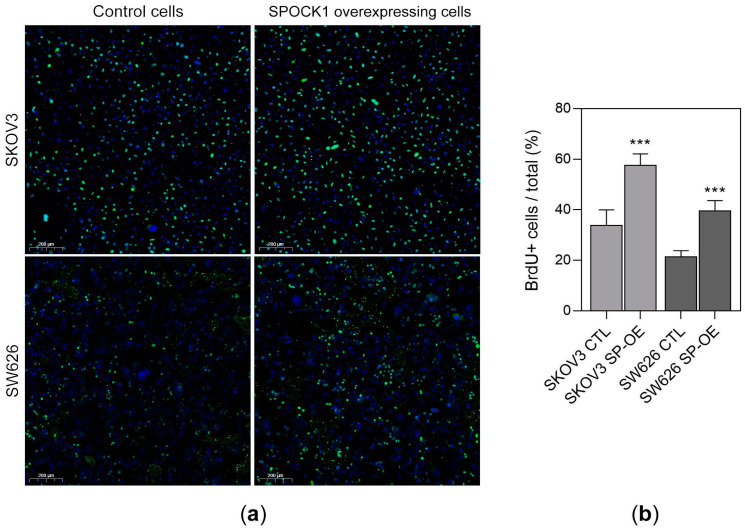
BrdU incorporation assay performed on SKOV3 and SW626 cells. (**a**) Fluorescent immunostaining specific for BrdU on SKOV3 and SW626 control and SPOCK1 overexpressing cells. Scale bar = 200 µm. (**b**) Quantitation of BrdU positive cells in SKOV3 control (SKOV3 CTL) and SPOCK1 overexpressing (SKOV3 SP-OE), as well as in SW626 control (SW626 CTL) and SW626 SPOCK1 overexpressing (SW626 SP-OE) cell lines. Results are expressed as mean ± SEM. *** *p* < 0.001.

To test whether SPOCK1 overexpression has any effect on DNA synthesis and proliferation, a BrdU incorporation assay was performed on both ovarian cancer cell lines (Figure 5a,b). It was found that 57.6% of SPOCK1 transfected SKOV3 cells were positive for BrdU staining, compared to 33.8% of the SKOV3 control cells (*p* < 0.001). Similarly, 39.5% of SW626 SPOCK1 overexpressing cells exhibited BrdU positivity, while only 21.4% of control SW626 cells displayed fluorescence (*p* < 0.001) (Figure 5b).

The significantly increased incorporation of BrdU provoked by SPOCK1 overexpression reflects the stimulated DNA synthesis (thus proliferation) in both ovarian cancer cell lines.

### 3.5. The Impact of SPOCK1 on Tumor Cell Migration

**Figure 6:**
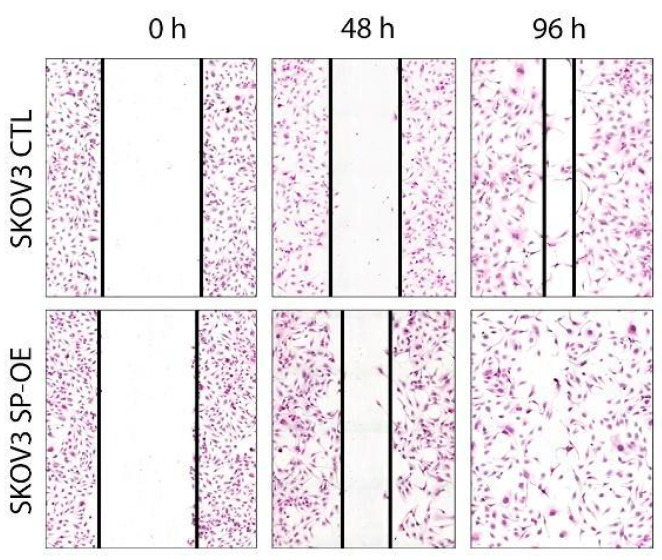
Wound healing assay of SKOV3 control (CTL) and SPOCK1 transfected (SP-OE) cells.

As a next step, we tested whether SPOCK1 exerts any effect on the migration of ovarian cancer cell lines. A wound healing assay showed that SPOCK1 overexpression enhanced the migration of SKOV3 cells, as seen at the 48 h time point, and resulted in full closure of the scratch at the end of the experiment (96 h), in contrast to that of control cells (Figure 6). SW626 cells did not migrate in our experimental set-up. These results suggest that high SPOCK1 levels may contribute to the spread of tumors.

### 3.6. Tumor Promoting Effect of SPOCK1 via p21^CIP1^

**Figure 7:**
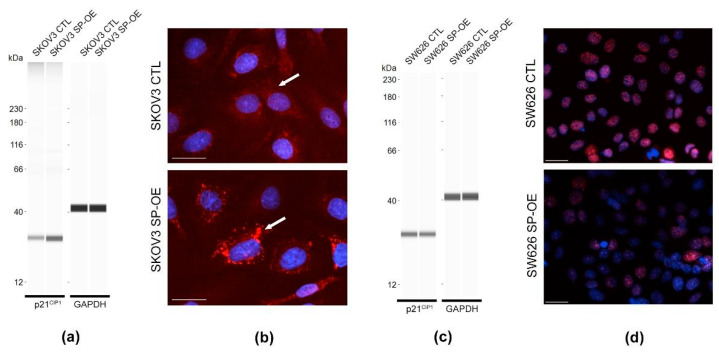
Detection of p21^CIP1^ tumor suppressor levels and localization upon SPOCK1 transfection in ovarian cancer cell lines. (**a**) WES analysis performed on control (CTL) and SPOCK1 overexpressing (SP-OE) SKOV3 cell lysates. (**b**) P21^CIP1^ immunostaining performed on SKOV3 control and transfected cells. Arrows indicate the staining intensity detected in the cytoplasm. P21^CIP1^ WES analysis (**c**) and immunostaining (**d**) conducted on SW626 control and SPOCK1 transfected cells. Scale bars: 50 µm.

As p21^CIP1^ cyclin-dependent kinase inhibitor is one of the main regulators of the cell cycle, its level and localization was analyzed by WES and immunostaining (Figure 7). Surprisingly, the protein level of the tumor suppressor was elevated in SPOCK1-overexpressing SKOV3 cell lysates compared to the control (Figure 7a). However, immunostaining revealed that the excess p21^CIP1^ was retained in the cytoplasm (Figure 7b). In SW626 cells, SPOCK1 transfection resulted in lower p21^CIP1^ levels than those of control cells, as seen in the WES analysis (Figure 7c). Here, canonical nuclear staining was detected in both control and SPOCK1 overexpressing cells, showing much less intensity in the latter, reflecting the enhanced cell cycle progression (Figure 7d).

### 3.7. SPOCK1 Serum and Tissue Levels in Patients with Ovarian Cancer

**Figure 8:**
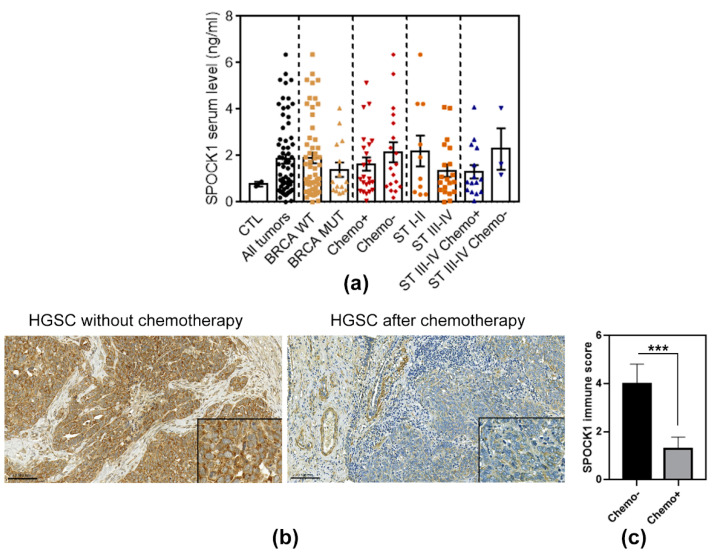
SPOCK1 plasma and tissue levels in patients with ovarian cancer. (**a**) SPOCK1 concentration measured by ELISA. Data are expressed as mean ± SEM. CTL: control, WT: wild type, MUT: mutant, Chemo+: received chemotherapy, Chemo-: not received chemotherapy ST: stage. (**b**) SPOCK1 immunostaining performed on high-grade serous carcinomas (HGSCs) prior and after chemotherapy treatment. Scale bar: 100 µm, and 50 µm for inserts. (**c**) Quantification of SPOCK1 staining intensity in chemotherapy-treated (Chemo+) and non-treated (Chemo−) tumor tissues. Results are expressed as mean ± SD, *** *p* < 0.001.

As reported above, in SKOV3 cells, the vast majority of excess SPOCK1 was secreted to the culture medium. We therefore collected blood samples from patients with ovarian cancer and measured the SPOCK1 level by ELISA (Supplementary Table S3). In the plasma of cancer patients, the proteoglyan concentration was ~1.85 nm/mL, while ~0.77 ng/mL was measured in the control plasma of healthy individuals (Figure 8a). Interestingly, its level seems to be associated with BRCA mutation, exhibiting lower levels in patients harboring mutations. Surprisingly, higher plasma concentrations were measured in patients with tumors who had not received any chemotherapy, compared to the serum from chemotherapy-treated individuals (~2.15 ng/mL vs. ~1.64 ng/mL) (Figure 8a). When staging was taken into account, higher SPOCK1 levels were detected in stage I and II tumors compared to stage III and IV (~2.2 ng/mL vs. ~1.35 ng/mL). However, when stage III-IV tumors were divided into treated and non-treated groups, again, the difference between chemotherapy non-receivers and receivers (~2.3 ng/mL vs. ~1.3 ng/mL) became apparent (Figure 8a) suggesting an association between SPOCK1 serum levels and chemotherapy treatment.

To validate our ELISA results, SPOCK1 was immunostained again in FFPE tumor tissue samples to measure any differences associated with chemotherapy (Figure 8b). Ten samples were taken from tubo-ovarian high-grade serous carcinomas (HGSCs) removed before any treatment, and 10 samples derived from HGSCs removed during delayed debulking surgery, after the application of neoadjuvant paclitaxel and carboplatin treatment. In all pre-treatment cases of HGSCs, the tumor cells showed the same diffuse granular cytoplasmic SPOCK1 staining with strong or moderate intensity as seen before, with a mean immune score of 4 (Figure 8c, Table S2). In the samples taken after neoadjuvant treatment, however, a significant decrease was observed in SPOCK1 expression in tumor cells, with a mean immune score of 1.3 (*p* < 0.001) (Figure 8c, Table S2). In all samples, the majority of tumor cells still expressed SPOCK1, but the staining intensity was milder, compared to the smooth muscle cells of vessels.

### 3.8. SPOCK1 Expression Is Related to the CHD1L Transcription Factor

**Figure 9:**
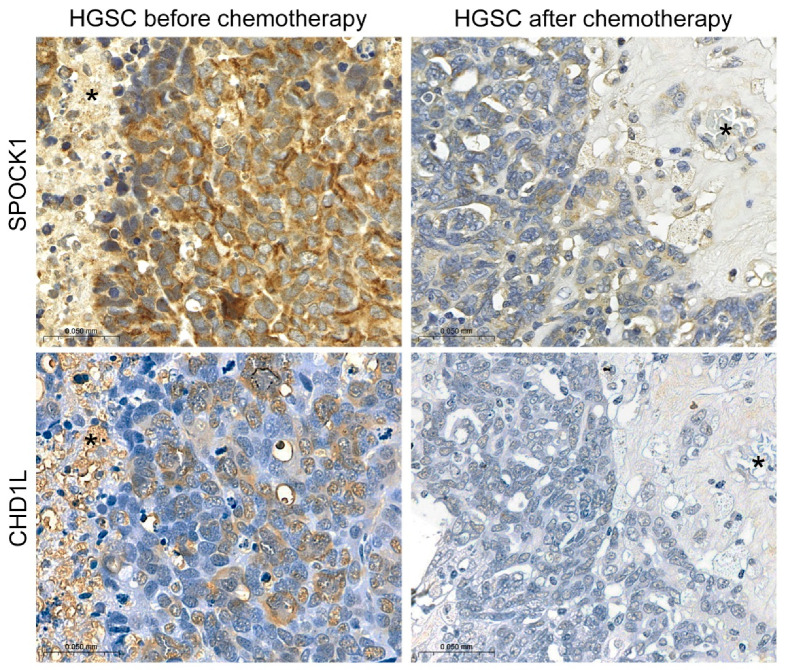
Immunostaining of CHD1L transcription factor and SPOCK1 in ovarian cancer with or without chemotherapy treatment. Scale bar: 100 µm. Asterisks mark the same structures on serial sections.

The decreased SPOCK1 expression related to chemotherapy raised the question of whether the inhibition appears at transcriptional level. Thus, chromodomain-helicase-DNA-binding protein 1-like (CHD1L), a known transcription factor for SPOCK1, was immunostained in parallel with SPOCK1 on the same tissue samples (Figure 9). In tumors that had not been treated with chemotherapy, where SPOCK1 exerted strong staining, tumor cell nuclei were also positive for CHD1L. On the other hand, chemotherapy-treated tumor samples with weak SPOCK1 expression were negative for CHD1L, suggesting that the regulatory mechanism of SPOCK1 may appear at mRNA level.

## 4. Discussion

There are an increasing number of published studies discussing the oncogenic role of SPOCK1 in a variety of malignancies; however, studies exploring its implications in ovarian cancer, especially using human material, are still scarce. Information can be extracted from comprehensive studies (mostly in silico), although, so far, only one publication has investigated the influence of SPOCK1 specifically in ovarian cancer [17]. Therefore, we aimed to examine SPOCK1′s role in ovarian cancer in further detail. To this end, in vitro SKOV3 and SW626 cell lines were transfected with vectors overexpressing SPOCK1, in order to assess its effect on the behavior of tumor cells. Human FFPE biopsies were utilized to evaluate SPOCK1 protein levels and localization in tumors and normal ovaries, and blood samples were collected to determine serum levels of the proteoglycan in ovarian cancer patients.

Members of the SPARC protein family, including SPOCK1 [19], are matricellular proteins with no structural function but multiple roles in maintaining a healthy ECM and in the interplay between ECM and cellular compartments via integrins or other surface receptors (e.g., EGFR or endoglin) [20,21]. SPOCK proteins possess GAG chains, allowing them to interact not only via their protein core, but also through their sugar chains. All three members of the Testican/SPOCK family have a similar 5-domain structure [22]. However, the literature provides little information regarding their structural properties or changes.

The SPOCK1 gene has ten splice variants, of which three isoforms are predominantly expressed in all cancers [12]. In ovarian cancer, SPOCK1-201 and SPOCK1-001 mainly occur, as well as small amounts of SPOCK1-003 [12]. According to the Ensembl database, only four of the splice variants have a protein product and only two of them carry signal peptide, allowing the protein to be secreted. The largest protein core synthesized is 49.1 kDa, hence all structural variants identified in SKOV3 and SW626 cells must be glycanated, however, the extent and structure of the glycanation remains unknown. SPOCK1 is recognized to be a modular proteoglycan with two GAG attachment sites for HS [23], but the two different cell lines, SKOV3 and SW626, manufacture SPOCK1 of various sizes. The 72 kDa form has been described, not only in ovarian cells, but also in liver cancer cell lines [15]. Transfection with the identical SPOCK1 overexpressing vector resulted in overproduction of different SPOCK1 forms in the two cell lines, suggesting distinct regulatory mechanisms. Untransfected wild type cells do not secrete SPOCK1 into their culture medium. When transfected with SPOCK1, only SKOV3 cells transported the proteoglycan to the medium in a highly glycosylated form that was not present within the cell. Doubts remain as to whether this is the same isoform as that found within the cytoplasm, or a different type containing a signal peptide. However, it is known that glycosylation level, GAG length and the structure of proteoglycans can alter during various pathological events.

Although numerous publications have reported that SPOCK1 promotes the proliferation and migration of cell lines derived from different types of tumors [10,11,24,25,26,27,28,29,30,31,32], only one study has demonstrated that silencing SPOCK1 attenuated proliferation, migration and invasion of ovarian cancer cell lines [17]. In our set of experiments, we aimed to determine whether SPOCK1 overproduction stimulates proliferation and migration of SKOV3 and SW626 cell lines. As anticipated, transfection with a vector overexpressing SPOCK1 caused both cell lines to exhibit significantly increased precursor uptake for DNA synthesis, as measured by BrdU assay and, in the case of the SKOV3 cell line, the excessive proteoglycan production also stimulated cell migration. Although examining the signaling network affected by SPOCK1 transfection was not the purpose of our study, we did see alterations in the level of a crucial tumor suppressor, p21^CIP1^.

Transfection of SPOCK1 into SKOV3 cells prevented p21^CIP1^ from entering the nucleus, resulting in cytoplasmic accumulation. Phosphorylation is a known retention mechanism for p21^CIP1^ in the cytoplasm [33]. AKT/PKB, which enhances the stability and cytoplasmic localization of p21^CIP1^ and promotes cell survival, is one of the major molecules responsible for phosphorylating p21^CIP1^ [34]. Numerous studies indicate that SPOCK1 exerts its oncogenic action via the PI3K/AKT/mTOR pathway [11,26,29,30,31,35,36]. Therefore, we can assume that SPOCK1 overexpression in SKOV3 cells activates the AKT pathway, resulting in an increase in the phosphorylation of p21^CIP1^ and its subsequent accumulation in the cytoplasm. Intriguingly, cytoplasmic p21^CIP1^ levels were found to predict cisplatin resistance in human testicular cancer [37] and cisplatin sensitivity in ovarian cancer [38]. Based on these data, it is conceivable that p21^CIP1^ is also involved in the link between SPOCK1 levels and chemotherapy treatment. In SW626 cells, p21^CIP1^ operated in a conventional manner, as it was localized in the nucleus and its level fell in parallel with the higher DNA synthesis rate in SPOCK1 transfected cells [39].

In tumor tissues, the tumor cells mainly exhibited moderate or strong diffuse granular cytoplasmic SPOCK1 staining. The granular staining seen for SPOCK1 in our study has been previously documented in liver tumor cells [15,22], where the proteoglycan colocalized with mitochondrial markers. Given the similar pattern observed in ovarian carcinomas, it is possible that SPOCK1 resides in the mitochondria.

Only one publication has so far reported the presence of SPOCK1 in serum samples from patients with sepsis [16]. Here, we report for the first time that SPOCK1 is detectable in the blood samples of patients suffering from ovarian cancer, and that its level may correlate with chemotherapy treatment. Despite the fact that our cohort was relatively small and grouping further reduced the number of patients, immunostaining performed on selected samples with or without chemotherapy treatment confirmed the ELISA results, as all treated tumor samples exhibited significantly lower SPOCK1 levels than those of non-treated tumor samples.

Regarding chemotherapy, a number of studies have implicated SPOCK1 in the resistance of tumor cells to various drugs. SPOCK1 plays a significant role in the establishment of a phenotype of colorectal cancer cell resistant to 5-FU. In line with this, SPOCK1 upregulation was associated with advanced stages of CRCs [40]. In addition, SPOCK1 has been shown to be involved in tumor metabolism such as glucose consumption and lactic secretion and to promote the Warburg effect [41]. These findings suggest that targeting SPOCK1 may be a potential therapeutic method to overcome 5-FU resistance in CRC. In lung cancer, SPOCK1 expression was elevated in osimertinib-resistant tumor cells, and its knockdown inhibited the proliferation of osimertinib-resistant cells and overcame resistance. Research has identified SPOCK1 as an independent prognostic factor in NSCLC, with the potential to become a target gene for therapy in osimertinib-resistant lung cancers [42]. Glioblastoma and gastric cancer are two further instances of SPOCK1 involvement in chemotherapy. In recurrent glioblastoma tumors, the proteoglycan was highly expressed, and contributed to metastasis formation and temozolomide resistance [43]. In gastric cancers, acquired lapatinib resistance was mediated by SPOCK1-induced EMT [44].

All of the aforementioned studies demonstrated that SPOCK1 is involved in the development of resistance to certain chemotherapeutics, and that targeting SPOCK1 can reverse the process. However, none suggested that the efficacy of chemotherapy could be inferred by monitoring SPOCK1 levels. We propose that, based on our findings on tumor tissue materials and serum samples, SPOCK1 may be a candidate for detecting the efficacy of chemotherapy, as its level decreases in samples from patients who have received chemotherapy.

Chromodomain-helicase-DNA-binding protein 1-like (CHD1L) is regarded as an oncogene that participates in transcriptional regulation, maintenance of chromosome integrity and DNA repair. Among others (such as ARHGEF9 (Rho guanine nucleotide exchange factor 9 and TCTP (translationally controlled tumor protein)), it functions as a transcription factor for SPOCK1 by binding directly to its promoter region [26,45]. Through activation of the AKT signaling pathway, inhibition of cytochrome c release and activation of caspase-3 and -9, CHD1L-mediated overexpression of SPOCK1 prevents apoptosis of HCC cells. Using AKT inhibitors, these effects are suspended. In addition, HCC cells with elevated SPOCK1 expression have higher matrix metallopeptidase 9 levels; these cells were found to be more invasive and generated more metastatic nodules in vivo than HCC cells with lower SPOCK expression [26]. CHD1L protein is overexpressed in human ovarian carcinomas and serves a prognostic biomarker for patients’ survival [46]. We detected higher CHD1L levels in ovarian cancer samples that had not received treatment, but its expression vanished in samples from patients receiving chemotherapy. We do not yet know whether there is a direct functional link between SPOCK1 and CHD1L expression in ovarian cancer, and thus more research is required to elucidate the issue.

In general, SPOCK1 expression is higher in tumors than in their non-tumorous adjacent tissues, as determined by a comprehensive analysis across different types of cancers, and its overexpression has been linked to poor clinical outcomes [12]. By analyzing gene expression and tumor-infiltrating immune cells, the same study revealed that SPOCK1 level was correlated with several immune cells across cancers [12]. Alone or as part of gene signatures, SPOCK1 has been associated with poor survival in various cancer types. In pancreatic cancer, for instance, high levels of the proteoglycan were correlated with shorter overall and disease-free survival, both as part of a gene signature and independently [25,47]. Similarly, it served as a prognostic factor as a component of a 6-gene EMT signature in lung cancer [48]. Bioinformatic analysis of clear cell renal cell carcinoma revealed that SPOCK1 mRNA was elevated in tumor tissues relative to non-tumorous adjacent tissues, and that higher SPOCK1 expression was associated with shorter survival and correlated with infiltration of tumor-associated fibroblasts and macrophages, suggesting a potential prognostic biomarker [49]. In prostate cancer, high SPOCK1 expression was related to advanced stage, T value and Gleason grade. Similarly to other cancers, tumor tissues had higher SPOCK1 levels than non-tumor tissues, and PaC patients with a high SPOCK1 expression had a worse median overall and disease-free survival [50,51].

In ovarian cancer, the Protein Atlas Database clearly revealed that high SPOCK1 expression is a factor associated with poor prognosis and reduced survival rate. However, Kaplan–Meier plotter analysis showed that the proteoglycan level has the greatest influence on tumors of grades I and II, whereas the SPOCK1 level had minimal effect on grade III tumors. It is understandable, given the association between the proteoglycan and chemotherapy, that patients with more advanced ovarian tumors practically always undergo chemotherapy. Consequently, additional research is required on a stratified patient cohort in relation to chemotherapy.

## 5. Conclusions

The SPOCK1 gene has been identified as an oncogene involved in key cellular processes, including cell cycle control, DNA repair, apoptosis and metastasis. Through the EMT process, it impacts the migration and invasion of cancer cells in a number of cancers. It has been demonstrated that overexpression of the SPOCK1 gene promoted the development and progression of tumors and is associated with shorter survival rates, indicating that it may become a viable anti-tumor therapeutic target. According to the findings of the present study, all of the aforementioned statements are accurate for ovarian cancer. In addition, the SPOCK1 level appears to be associated with chemotherapy treatment and might be used for monitoring the disease.

## Data Availability

The original contributions presented in the study are included in the article/Supplementary Material. Further inquiries can be directed to the corresponding author.

## Supplementary Materials

The following supporting information can be downloaded at: https://www.mdpi.com/article/10.3390/cancers15072037/s1, Table S1: Antibodies used in the present study; Table S2: Evaluation of SPOCK1 immunostaining on human tissue samples. Table S3: Patient details and raw data of SPOCK1 plasma levels.
