# Supplementary material for: SPOCK1 Overexpression Suggests Poor Prognosis of Ovarian Cancer
Source: Cancers (Basel). 2023 Mar 29;15(7):2037. doi: 10.3390/cancers15072037 (PMC10093273; doi:10.3390/cancers15072037)
Supplement: Supplementary file 1 [file cancers-15-02037-s001.zip › cancers-2223419-supplementary.pdf]

Supplementary material

Supplementary Table S1. Antibodies used in the present study

**Primary Antibody**	**Host species, isotype**	**Manufacturer**	**Cat. No.**	**Dilution**		
				**IHC**	**ICC**	**WES**
**Anti-BrdU**	Mouse, monoclonal, (clone B44)	Becton, Dickinson and Company BD, Franklin Lakes, NJ USA	347580		1:50	
**CHD1L**	Rabbit, polyclonal	Merck KGaA, Darmstadt, Germany	HPA028670	1:1000		
**Cross-Adsorbed Secondary Antibody, Alexa Fluor 568**	Goat anti-Rabbit IgG	Invitrogen, CA, USA	A11011		1:200	
**GAPDH**	Rabbit, monoclonal (clone 14C10)	Cell Signaling Technology, Danvers, MA, USA	2118			1:50
**p21**	Rabbit, monoclonal (clone EPR18021)	Abcam, Cambridge, UK	ab188224			1:1000
**Spock1**	Rabbit, polyclonal	Merck KGaA, Darmstadt, Germany	HPA07450	1:100	1:200	1:50

Supplementary Table S2. Evaluation of SPOCK1 immunostaining on human tissue samples.

Samples	Chemotherapy	SPOCK1 staining internsity
S1	No	3
S2	No	4
S3	No	4
S4	No	3
S5	No	4
S6	No	4
S7	No	5
S8	No	3
S9	No	5
S10	No	5
S11	Yes	2
S12	Yes	1
S13	Yes	2
S14	Yes	1
S15	Yes	1
S16	Yes	1
S17	Yes	1
S18	Yes	1
S19	Yes	1
S20	Yes	2
S21	N/A	5
S22	N/A	1
S23	N/A	3
S24	N/A	5
S25	N/A	4

Supplementary Table S3. Patient details and raw data of SPOCK1 plasma levels

Patient ID	Age	Diagnosis (SNOMED)	BRCA status	Chemotherapy	Stage	SPOCK1 level (ng/ml)
P1	74	Malignant tumor of ovary (363443007)	WT	N/A	N/A	1,179
P2	65	Malignant tumor of ovary (363443007)	mut	N/A	N/A	0,876
P3	68	Malignant tumor of ovary (363443007)	WT	N/A	N/A	3,199
P4	54	Malignant tumor of ovary (363443007)	WT	N/A	N/A	0,788
P5	62	Malignant tumor of ovary (363443007)	mut	Yes	N/A	2,634
P6	70	Malignant tumor of ovary (363443007)	WT	No	N/A	0,187
P7	60	Malignant tumor of ovary (363443007)	mut	N/A	N/A	0,420
P8	84	Malignant tumor of ovary (363443007)	WT	No	N/A	1,449
P9	79	Malignant tumor of ovary (363443007)	WT	Yes	IV	0,433
P10	71	Malignant tumor of ovary (363443007)	mut	Yes	N/A	0,470
P11	72	Malignant tumor of ovary (363443007)	WT	No	N/A	2,103
P12	61	Malignant tumor of ovary (363443007)	WT	No	N/A	0,489
P13	80	Malignant tumor of ovary (363443007)	WT	No	N/A	5,517
P14	51	Malignant tumor of ovary (363443007)	mut	N/A	I	2,486
P15	83	Malignant tumor of ovary (363443007)	WT	Yes	IV	0,986
P16	61	Malignant tumor of ovary (363443007)	WT	Yes	III	0,355
P17	61	Malignant tumor of ovary (363443007)	mut	Yes	III	0,525
P18	64	Malignant tumor of ovary (363443007)	WT	N/A	N/A	0,296
P19	79	Malignant tumor of ovary (363443007)	WT	No	IV	1,629
P20	74	Malignant tumor of ovary (363443007)	WT	No	N/A	0,464
P21	44	Malignant tumor of ovary (363443007)	WT	N/A	I	0,333
P22	66	Malignant tumor of ovary (363443007)	WT	Yes	II	1,000
P23	61	Malignant tumor of ovary (363443007)	mut	N/A	N/A	0,378

P24	68	Malignant tumor of ovary (363443007)	WT	N/A	III	0,637
P25	42	Malignant tumor of ovary (363443007)	WT	N/A	II	0,432
P26	42	Malignant tumor of ovary (363443007)	WT	No	II	6,353
P27	62	Malignant tumor of ovary (363443007)	WT	N/A	N/A	0,947
P30	28	Sine morbo	mut	No	N/A	0,876
P31	34	Sine morbo	mut	No	N/A	0,678
P28	47	Malignant tumor of ovary (363443007)	WT	N/A	III	0,000
P29	56	Malignant tumor of ovary (363443007)	WT	N/A	N/A	0,875
P30	63	Malignant tumor of ovary (363443007)	WT	N/A	I	0,326
P31	76	Malignant tumor of ovary (363443007)	WT	N/A	N/A	0,554
P32	71	Malignant tumor of ovary (363443007)	WT	N/A	N/A	4,462
P33	49	Malignant tumor of ovary (363443007)	mut	N/A	N/A	1,758
P34	69	Malignant tumor of ovary (363443007)	WT	N/A	II	1,932
P35	84	Malignant tumor of ovary (363443007)	WT	N/A	I	4,231
P36	56	Malignant tumor of ovary (363443007)	mut	No	II	0,660
P37	48	Malignant tumor of ovary (363443007)	WT	Yes	IV	0,442
P38	60	Malignant tumor of ovary (363443007)	mut	No	N/A	3,399
P39	53	Malignant tumor of ovary (363443007)	mut/wt	N/A	N/A	0,508
P40	68	Malignant tumor of ovary (363443007)	WT	Yes	IV	0,843
P41	59	Malignant tumor of ovary (363443007)	WT	Yes	IV	0,048
P42	59	Malignant tumor of ovary (363443007)	WT	N/A	N/A	1,929
P43	55	Malignant tumor of ovary (363443007)	WT	N/A	N/A	4,480
P44	55	Malignant tumor of ovary (363443007)	WT	N/A	N/A	1,525
P45	71	Malignant tumor of ovary (363443007)	WT	Yes	IV	2,699
P46	57	Malignant tumor of ovary (363443007)	WT	Yes	III	4,081
P47	51	Malignant tumor of ovary (363443007)	mut	No	III	1,172

P48	84	Malignant tumor of ovary (363443007)	WT	Yes	N/A	2,084
P49	59	Malignant tumor of ovary (363443007)	WT	Yes	N/A	0,748
P50	55	Malignant tumor of ovary (363443007)	WT	No	N/A	0,778
P51	56	Malignant tumor of ovary (363443007)	WT	Yes	N/A	1,740
P52	71	Malignant tumor of ovary (363443007)	WT	Yes	III	0,826
P53	66	Malignant tumor of ovary (363443007)	WT	N/A	N/A	0,386
P54	54	Malignant tumor of ovary (363443007)	mut	No	III	4,045
P55	67	Malignant tumor of ovary (363443007)	WT	Yes	IV	0,862
P56	53	Malignant tumor of ovary (363443007)	WT	N/A	N/A	5,257
P57	68	Malignant tumor of ovary (363443007)	WT	No	N/A	3,761
P58	30	Malignant tumor of ovary (363443007)	WT	N/A	N/A	1,899
P59	57	Malignant tumor of ovary (363443007)	WT	No	N/A	2,763
P60	52	Malignant tumor of ovary (363443007)	WT	Yes	N/A	5,133
P61	71	Malignant tumor of ovary (363443007)	WT	Yes	IV	2,328
P62	75	Malignant tumor of ovary (363443007)	WT	No	N/A	2,339
P63	52	Malignant tumor of ovary (363443007)	WT	Yes	I	4,224
P64	62	Malignant tumor of ovary (363443007)	WT	Yes	IV	2,479
P65	72	Malignant tumor of ovary (363443007)	WT	Yes	IV	1,083
P66	74	Malignant tumor of ovary (363443007)	WT	Yes	IV	1,588
P67	64	Malignant tumor of ovary (363443007)	mut	N/A	N/A	3,663
